# The Association between Medication Experiences and Beliefs and Low Medication Adherence in Patients with Chronic Disease from Two Different Societies: The USA and the Sultanate of Oman

**DOI:** 10.3390/pharmacy9010031

**Published:** 2021-02-03

**Authors:** Kamla M. Ibrahim, Jon C. Schommer, Donald E. Morisky, Raquel Rodriguez, Caroline Gaither, Mark Snyder

**Affiliations:** 1 Department of Pharmaceutical Care & Health Systems, College of Pharmacy, University of Minnesota, Minneapolis, MN 55455, USA; schom010@umn.edu (J.C.S.); rodre001@umn.edu (R.R.); cgaither@umn.edu (C.G.); 2 Community Health Sciences, Fielding School of Public Health, University of California, Los Angeles, CA 90095, USA; dmorisky@ucla.edu; 3 Center for the Study of the Individual and Society, Department of Psychology, College of Liberal Arts, University of Minnesota, Minneapolis, MN 55455, USA; msnyder@umn.edu

**Keywords:** medication experiences, belief, medication adherence, Oman

## Abstract

This cross-sectional study aimed to describe the association between medication experiences and beliefs and self-reported medication adherence in patients with chronic diseases in two different samples from two different societies: the USA and the Sultanate of Oman. The Morisky Medication Adherence Score (MMAS-8) questionnaire was used to measure medication adherence. Three items (statements) were used for measuring medication experiences and beliefs variable on a four-point Likert scale adapted from the 2015 National Consumer Survey of the Medication Experience and Pharmacists’ Role (NCSME&PR). In the U.S., quantitative secondary data analysis of 13,731 participants was conducted using the 2015 NCSME&PR, a self-administered online survey coordinated by Qualtrics Panels between 28 April 2015 and 22 June 2015. The same variables were translated into Arabic, with studies conducted at the Royal Court Medical Center in Oman, and data from 714 participants were collected between 16 June 2019 and 16 August 2019. Data were analyzed using IMB/SPSS version 24.0 software. Chi-square analysis and descriptive statistics were used. The results showed that the low adherence rates for medication (MMAS-8 < 6) were 56% and 52% in Omani and U.S. groups, respectively. Approximately 90% of the U.S. and Omani participants believed that “medicines are a life-saver”; however, medication adherence was higher in Oman (30%) than in the United States (9%) for these participants. In total, 60% of the U.S. and 29% of Omani participants believed that “medicines are a burden”; however, about 60–65% of participants in both countries were in the low medication adherence group. Additionally, 63% of the U.S. and 83% of the Omani participants disagreed that “medicines do more harm than good”; however, medication adherence in the U.S. (15%) was higher than in Oman (8%). In conclusion, a decrease in low medication adherence was observed with positive medication experiences and beliefs. However, the impacts of medication experiences and beliefs on low medication adherence rates were different from one population to another. The “medication burden” statement resulted in the highest percentage of difference in terms of low medication adherence rates between those who agree and those who disagree in the U.S. group (20%), whereas the “medicines are a life-saver” statement resulted in a greater difference in the Omani group (30%). Proper communication between patients and healthcare providers based on the patient’s medication experiences and beliefs will substantially improve medication adherence.

## 1. Introduction

Medication adherence, which is defined as “the degree to which a person’s behavior corresponds with the agreed recommendations from a healthcare provider” [1], has been one of the most central topics in healthcare for decades and is still the case today [2,3,4]. Non-adherence negatively impacts the health of millions of Americans and costs billions of dollars annually. In the United States, around 3% to 10% of avoidable healthcare costs annually ($100–$300 billion) are attributed to non-adherence [5]. About 133 million people, representing more than 40% of the U.S. population, have one or more chronic diseases [6]. Six out of 10 have a chronic condition, and four out of 10 have more than one chronic disease [7]. Seven out of 10 deaths in the U.S. are due to chronic diseases [8]. Heart disease, diabetes, breathing problems, cancer, stroke, and arthritis are the most common chronic diseases in the United States [7]. Adherence to medications for treating chronic conditions is a significant problem in healthcare, which has substantially impacted clinical outcomes and healthcare expenditure.Treating people with chronic diseases costs around 90% ($3.5 trillion) of the nation’s healthcare costs annually [7]. Nationally, spending on out-patient medication has increased by up to 18% per year over the past few years [9]. Nevertheless, medication adherence is generally low, at around 50% to 65% on average [10,11].

Many factors, such as the nature of the disease; the treatment regimen or complexity; and the side effects, financial burden, and patient behavior for a specific type of disease can all affect adherence [12,13,14]. These factors may shape the individual’s experiences with medication and may influence their medication beliefs. Furthermore, the medication experience is a social and personal experience shaped by a person’s traditions, religion, culture, life experiences, and what they have learned from others [15,16]. Individuals’ beliefs about health and illness and their behavior based on their medication experience are essential contributors to their adherence behavior [17,18]. This reflects patients’ decision-making and medication-taking behaviors, which affect their medication adherence [19]. Therefore, knowing patients’ medication experiences and beliefs may help healthcare providers understand and determine how and why patients decide whether to adhere to their medications.

This study intends to help healthcare providers find another substantial way to improve patients’ medication adherence by developing a tool for counseling patients with chronic disease according to their medication experiences and beliefs. Medication experiences and beliefs may differ from one society to another, according to a society’s culture, traditions, and religion. No study we know of has directly compared how the medication experience and belief concepts can differ from one society to another and have different effects on medication adherence from one society to another. Therefore, this is a one-of-a-kind study that identifies the association between medication experiences and beliefs and medication adherence in patients with different chronic diseases in two different populations: the USA and the Sultanate of Oman, with the same study variables. Furthermore, only two studies have been conducted in Oman, with small sample sizes (45 and 215 participants), to assess the relationship between hypertensive patients’ medication adherence and their health beliefs [20,21]. Therefore, this study provides more information and insights about the association between medication experiences and beliefs and medication adherence in different chronic diseases with a larger sample size.

### 1.1. Significance

The term “adherence” comes from the Latin word “adhaerere”, which means to cling to, keep close, or remain constant [18]. The Oxford English Dictionary defines adherence as “attachment or commitment to a person, cause, or belief” [4]. The World Health Organization defines a patient’s adherence as “the extent to which a person’s behavior—taking medication—corresponds with agreed recommendations from a healthcare provider” [1]. It is clear from these definitions that taking full advantage of effective medications can be achieved if the patient is committed to following the prescribed treatment regimen with reasonable care. Furthermore, the patient’s determination is necessary to maintain a therapeutic regimen. 

The adherence rate is the percentage of the prescribed doses of medication taken by the patient over a specified period or data on dose-taking per day (taking the prescribed number of pills each day), and the timing of dose-taking (taking pills within the prescribed period) [22]. In acute disease conditions, patient medication adherence rates were typically higher than patients with chronic diseases; adherence rates among patients with chronic conditions were low and fell dramatically after the first six months of therapy [3,12,23,24]. For example, a retrospective cohort study including 34,501 participants showed that approximately half of the patients receiving statin therapy discontinued their medication within six months of starting the therapy, and many of them discontinued due to statin side effects [24]. 

The United States National Center for Health Statistics [25] defines chronic diseases as a disease that lasts for three months or more and cannot be prevented by vaccines, cured by medication, and does not simply disappear [25]. With this definition, we realize why it is difficult for patients to adhere to their medications [26]. Many factors affect medication adherence. For example, a study on the effects of dose frequency on medication adherence illustrated that patients with chronic diseases seem to be more adherent to a once-daily regimen than more frequently scheduled medication regimens [27]. Additionally, in comparison with medication adherence rates among patients with different chronic diseases, bivariate statistics and stratification analyses showed that 72% of individuals with hypertension had achieved adherence rates of 80% or better compared to those with hypothyroidism (68%), type 2 diabetes (65%), seizure disorders (61%), hypercholesterolemia (55%), osteoporosis (51%), or gout (37%) during the first year of drug therapy [11]. The treatment complexity, medication side effects, dose frequencies, and adverse reactions may negatively affect patients’ medication experiences and influence their medication beliefs.

Shoemaker (2008) defined medication experience as an individual’s subjective experience of taking medication in their daily life [19]. Studies showed that some people tend to view their medications as lifesavers that are necessary for their lives [15]. On the contrary, other people see their medications as a burden. Additionally, medication experiences and beliefs also reflect people’s opinions about the extent to which the drugs may cause harm compared to their benefits [8]. Positive patient expectations may advance adherence behavior [17]. In contrast, in one study, patients with coronary heart disease with a tendency to suppress emotional distress had a higher cardiac death rate [28].

Medication experiences of some side effects had a more significant impact than others on medication adherence. A study showed that about 86% of the hypertensive patients experienced medication side effects, and 34.5% became non-adherent [29]. In this study, excessive urination and decreased sexual drive significantly lowered medication adherence [29]. In another study, when conducting medication therapy management (MTM), pharmacists identified several examples of drug therapy problems associated with medication experience at the root [17]. 

Likewise, in Oman, a cross-sectional pilot study was conducted with 45 hypertensive patients to assess the relationship between medication adherence and health beliefs, using the health belief model [20]. Around 49% of participants were highly adherent to medication, and there was a significant association between medication adherence and health beliefs related to the necessity of the medication and perceived self-efficacy. More than half of the participants disagreed that their medicine disrupted their life (68.2%), taking medication worried them (57.8%), or they experienced having undesired side effects (51.1%). However, there was no relationship between concerns about antihypertensive medications and medication adherence. 

That pilot study was followed by another study of 215 hypertensive Omani patients using the Beliefs about Medicines Questionnaire (BMQ)-specific scale. It showed that participants with stronger beliefs about medication necessity were twice as likely to have high adherence (*OR* = 1.98, *p* = 0.006), and that participants who were concerned about their medication were about one-third as likely to have high adherence (*OR* = 0.34, *p* < 0.001) [21]. 

Thus, identifying which specific experiences affect patients the most can help resolve this and improve their adherence. Reducing medication burden by minimizing dose frequency or the number of pills (if possible) and adjusting to a patient’s lifestyle are examples. Overcoming potential medication harm by minimizing the side effects and drug-drug interactions is another example. All of the above examples will optimize positive patient medication experiences and beliefs and enhance their adherence.

Furthermore, medication experiences and beliefs can contribute to precision medicine goals, which are the treatment strategies and preventions that take individual variability into account [18]. This accounts for why patients accept or reject medical treatment, which influences their medication adherence. Additionally, as the United States’ healthcare system moves towards patient-centered care, patient medication experience and belief measurements and the Morisky Medication Adherence Scale (MMAS-8), which were used in this study, will contribute towards this patient-centered care approach, where self-reported measures will be used to assess a patient’s medication adherence from their point of view. 

### 1.2. Innovation

This study is in line with current research trends of drawing more attention to humans’ behavioral aspects that lead to better health outcomes. The outcomes will help provide more information to improve medication adherence through medication experiences and beliefs as an essential factor in achieving more effective patient–provider communication relationships. Improving communication skills will enhance healthcare providers’ counseling efficiency in information-giving and increase patients’ self-efficacy [30]. Medication experiences contribute to precision medicine and patient-centered care, which will improve medication adherence, and therefore overall health outcomes. Ultimately, this will reduce hospitalization rates and emergency visits [2] and will significantly lower overall healthcare costs and expenditures [31].

Moreover, this type of study was the first of its kind and size to be conducted in Oman, and at the Diwan of Royal Court Medical Services (the investigator’s workplace) in particular. It takes into consideration medication experiences and beliefs and other demographic information. A few studies were previously conducted in Oman to assess medication adherence; however, small sample size [20,21,32,33,34] was the main limitation. The Diwan of Royal Court Medical Services provides healthcare services to its employees and their families from different parts of the country. It maintains complete medical records and all patients’ medical history records, allowing for accurate and consistent findings.

### 1.3. Study Objective

This study’s objective was to describe the association between medication experiences and beliefs and self-reported medication adherence in patients with chronic disease in two different societies: the USA and the Sultanate of Oman. This goal was achieved by answering the following research questions:

1. Do medication experiences and beliefs affect medication adherence?
2. Do relationships between medication adherence and medication experiences and beliefs vary from one nation to another?

The findings will be applied to integrate individual medication experiences with patient care processes to improve healthcare quality and efficiency.

Detecting a difference in medication adherence due to a patient’s medication experiences and beliefs may help to improve their adherence [15,17,29]. 

## 2. Methodology

### 2.1. Overview

This study was based on existing survey data from the 2015 National Consumer Survey of the Medication Experience and Pharmacists’ Role (NCSME&PR), conducted by the University of Minnesota College of Pharmacy. The data were collected via an online, cross-sectional, self-administered survey coordinated by Qualtrics Panels in the United States, between 28 April 2015 and 22 June 2015. It consists of a wide variety of measurements (most commonly chronic disease prevalence in the USA; medication adherence, medication experiences and beliefs; and demographic variables such as age, education, race, ethnicity, and gender) and includes many vital variables to achieve the objectives of this paper. Data were obtained from 26,173 adult individuals aged above 18 years old and residing in the United States. The median age was 43 years old, and the female proportion was 71% of the respondents. At least 500 responses were received from each of the 50 states and the District of Columbia. Descriptive statistics from the survey dataset (frequencies, mean, median, mode, range) were computed to identify and resolve outliers, missing data, and other errors. 

None of the EQUATOR Network’s (CONSORT, STROBE, CONREQ, etc.) reporting guidelines applied to the proposed secondary data analysis approach. However, the Shanghai Archives of Psychiatry published a guideline for conducting a secondary analysis of existing data [35], which was used to guide this study.

The study design was based on the theoretical framework of the 2015 NCSME&PR. The specific objectives for the 2015 NCSME&PR were to identify and describe consumer segments based on relevant components of the medication experience. The 2015 NCSME&PR was based on a previous pilot study [36] conducted by the same authors (the principal investigator) of the 2015 and 2016 NCSME&PR [8,37]. One of the pilot study’s main goals was to collect initial data to describe respondents’ medication experiences. Medication belief structures reflect perceived necessity (viewing medications as life-savers that provide desired benefits), concern (viewing medications as life disruptors that are a reminder of illness and are a burden in their lives), and necessity–concern (viewing the overuse of medications and their potential for harm) viewpoints. The findings revealed that the medication experience is rooted in medication beliefs, personal abilities and motivations, information processing, decision-making, relationships, finances, and the effects of life experiences. The 2013 National Consumer Survey’s pilot study on medication experiences [36] showed that medication beliefs are reliable and valid constructs.

Based on the above, and with the view of that “a person’s medication experience is their personal approach to the use of medicines and is shaped by a person’s traditions, religion, culture, life experiences, and what they have learned from others” [16] and that “the medication experience is more than a clinical experience … it is a social and personal experience” [15], it was decided to apply the same survey to a different population, namely in Oman (the investigator’s home country), to determine whether the effects of medication experiences and beliefs on medication adherence are the same or different in a different society with different traditions, religion, culture, and life experiences.

Thus, based on the secondary data analysis findings of the 2015 NCSME&PR dataset and using the same variables, a new survey was developed, translated into the Arabic language, and conducted in the Sultanate of Oman. The Sultanate of Oman is an Arab Muslim country located on the Arabian Peninsula’s southeastern coast of Western Asia. It has a population of nearly 5 million, with 56% being Omani nationals and 43% expatriates. The average life expectancy of an Omani person is 73.8 years. Almost 70 percent of all Oman deaths are attributed to non-communicable diseases, at about 11 deaths/day. Approximately 36% of deaths are due to cardiovascular disease and high blood pressure, 12% to diabetes, 9% to cancer, and 2% to respiratory diseases [38].

Omani citizens have free access to public healthcare and free medication [39]. Oman has many public and private hospitals, public health centers, dispensaries, and private clinics around the country [39]. Health centers provide primary healthcare. The polyclinics provide secondary healthcare, and the hospitals provide tertiary and specialized care. Patients receive their medication from a pharmacy within a health center or hospital. 

**Figure 1:**
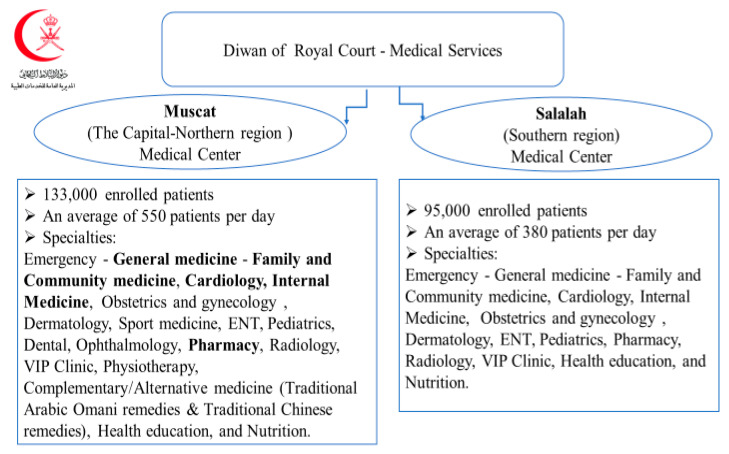
The health system of Diwan OF Royal Court Medical Services.

**Figure 2:**
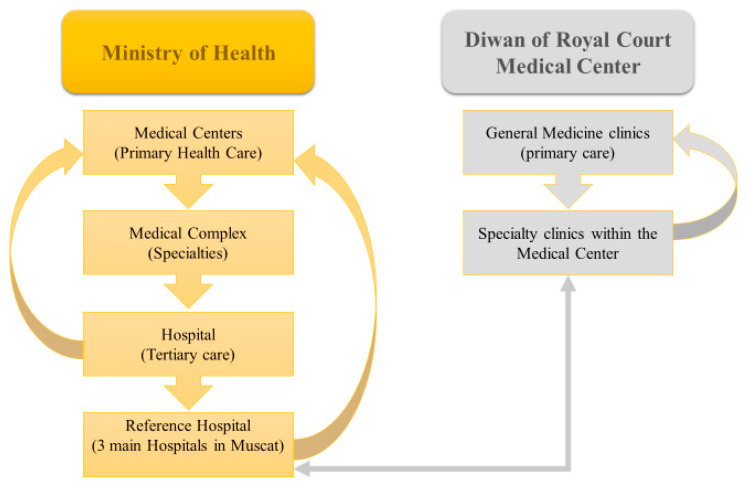
The health system of Oman and Diwan of Royal court Medical Center.

The Diwan of Royal Court Medical Services has two medical centers (Figure 1) that provide specialized medical services to the royal family and the employees of the Royal Court and their families [40]. The medical services of the Royal Court use a computerized system to record and store all patient-related information. Primary care physicians refer patients with chronic diseases to specialist clinics within the medical center. Once the patient has stabilized, they are followed again by the primary care physician (Figure 2). The pharmacy department provides all kinds of medications for various specialties, including all chronic diseases treated within the medical center. Patients with chronic diseases usually receive a two-month medication supply.

The United States health system is a mix of public and private insurers. Private health insurance is the greatest source of health insurance coverage for people younger than 65 years [41]. The individual either purchases health insurance or obtains it through employer-sponsored health insurance. For public insurance, the federal government provides a Medicare program for adults aged 65 and older and some people with disabilities. There are also various programs for veterans and low-income people, including Medicaid and the Children’s Health Insurance Program. The individuals choose hospitals or physicians who are covered by their insurance plan [41]. Health insurance plans often require individuals to choose a primary care physician upon enrollment who arranges referrals to specialist physicians if they need it. Patients go to the community pharmacy closest to their residence or any pharmacy they chose to dispense their prescription, covered by their health insurance plan, to get their prescribed medication.

Nevertheless, pharmacists carry out the same basic principles in dispensing prescriptions and counseling and educating patients in both countries. 

### 2.2. Design and Sample

A. Secondary data analysis: The United States

The preliminary analysis was conducted via secondary data analysis of the 2015 NCSME&PR. More details regarding the 2015 NCSME&PR are mentioned on the previous page (Overview). Eligibility for this secondary data analysis included adult patients above 18 years old who had or were taking one or more medications for chronic diseases. Thus, out of 26,173 responses, 13,731 participants were eligible for this study.

B. Data collection from a different population: The Sultanate of Oman

The same variables used in the preliminary analysis on the U.S. population were developed, translated, and conducted in the Omani population at the Diwan of Royal Court Medical Services.

I. Sampling Frame

The sampling frame included any adult patients taking at least one chronic disease medication and who were native Arabic language speakers registered in the Diwan Royal Court Medical Centers. Most patient responses were collected from Muscat Medical Center (in the capital city).

II. Sampling Method

The survey was collected manually from the waiting areas of the Internal Medicine Clinic, Cardiology Clinic, General Medicine Clinic, and pharmacy. The survey collection started from June 16 to 8 August 2019.

III. Sample Size

An online sample size calculator [42] was used to estimate the sample size. For a population of 133,000 [40] (number of patients registered at the Diwan of Royal Court Medical Services), there was a 95% level of confidence, 5% margin of error (the acceptable tolerability and most common margin of error is 5%), and 50% level of variance (as the studies mentioned above showed that medication adherence rates to chronic diseases were 50–60% on average). The sample size was estimated to be not less than 384 participants. However, 930 paper survey sets were distributed. The response rate was 76%. A total of 776 participant responses were collected.

IV. Inclusion Criteria

This survey’s eligibility included adult patients above 18 years old, taking at least one chronic disease medication, who were native Arabic language speakers and registered patients at the Diwan of Royal Court Medical Centers (either in Muscat or Salalah).

V. Exclusion Criteria

Patients who did not meet all four of the inclusion criteria were disqualified from participating in the survey.

VI. Process for Survey Development, Translation, and Data Collection

(a) Survey development process (Supplementary Material 1: Developed Survey)

1. The same variables (see Study Measures section) used in the secondary analysis were selected and a new survey was developed.
2. Element writing guidelines [43] and social exchange theory were applied to reduce the social desirability bias, reduce the measurement error, and increase the response rate.
3. The survey was reviewed by measurement expert Dr. Michael C. Rodriguez, a Professor of Quantitative Methods in Education, Campbell Leadership Chair in Education and Human Development, and Co-Director of the Educational Equity Resource Center at the Educational Psychology University of Minnesota. The measurement expert review aimed to ensure that the survey was well presented, increasing the response rate and reducing measurement errors.
4. The following copyright footnote was added in Arabic at the end of each page of the survey:

© جميع حقوق الطبع والتأليف والنشر للأسئلة من 2 إلى 9 في (ثانياً: أسئلة تتعلق بصحتك) هي محفوظة لـ Donald E. Morisky, ScD, ScM, MSPH, Professor, Department of Community Health Sciences, UCLA School of Public Health, 650 Charles E. Young Drive South, Los Angeles, CA 90095-1772, *dmorisky@ucla.edu.*

(b) Arabic translation process

The following steps were taken when translating the developed survey into the Arabic language:

1. The translation was done through a forward–backward translation process to ensure the questions were translated correctly. This process was performed by three individuals who speak Arabic from the Sultanate of Oman and who obtained their bachelor’s and master’s degrees from Western universities in English. The first person did the forward translation and then sent it to the second person who did the back translation. The third person was the investigator who modified the final version of the translation.
2. The Morisky Medication Adherence Scale (MMAS-8) [44,45,46,47] used to measure medication compliance was translated and validated by the scale’s inventor. The contract for the use of MMAS-8 stipulates that only the Arabic version provided by the inventor should be used.
3. Finally, the “think aloud” process was applied by two Arabic speakers to ensure that questions were answered correctly and that participants could understand and follow the instructions correctly (Supplementary Material 2: Arabic Translated Survey).
4. A pilot study of the first 80 paper survey sets was distributed at the three clinics and in the pharmacy waiting area. Some adjustments were made to the formatting and language of the questions. These adjustments reduced the number of unanswered questions and increased the overall response rate (for more details, see Data Collection Process).

(c) Oman approval process

Approval for the proposal and the use of the Arabic version of the survey was obtained from the Ethical Research Committee of Medical Services at the Diwan of Royal Court in Oman. The committee also approved the use of patient consent when conducting the survey (Supplementary Material 3: Consent Forms).

(d) Data Collection process

During the period from 16 June to 8 August 2019, data collection took place at the Diwan of Royal Court Medical Services in Muscat. Paper survey sets were distributed in the waiting areas of the Internal Medicine Clinic, Cardiology Clinic, General Medicine Clinic, and pharmacy. First, the nurse in charge at each clinic gave each patient eligible for the study the consent forms (see Supplementary Material 3) to complete or asked the patient verbally if they agreed to participate in the study. To ensure eligibility, the first page of the survey (see Supplementary Material 1) had two questions to be answered before starting to fill the survey.

The following observations were made:

1. The first 80 paper survey sets were distributed at the three clinics and the pharmacy waiting area as a pilot project. The nurse in charge of each clinic distributed paper scanning kits with pens to patients in the waiting area after completing the screening. In this pilot survey, 68 responders only answered the first few questions, and many others did not answer any questions at all. Therefore, each clinic’s nurses were asked to verify that the patient upon delivery answered all survey questions;
2. Many participants could not write or read, resulting in a lower response rate at the beginning of the data collection process. Therefore, three pharmacists and three nurses were appointed to assist any illiterate patient who was not accompanied by educated relatives. One pharmacist and one nurse were assigned to the Internal Medicine Clinic and the same in the General Medicine Clinic. Only one nurse was assigned to the Cardiology Clinic, as few patients were attending this clinic and it was open only twice a week. One pharmacist was also assigned in the pharmacy waiting area for any patient who may have missed the clinic’s questionnaire;
3. In the first week of the process, about 74 responses were discarded because participants missed many questions of the MMAS-8 questions. The investigator made some adjustments to the formatting and language of the questions. These adjustments reduced the number of unanswered questions and increased the overall response rate;
4. To ensure that the required sample size (500 or more) was obtained, the researcher examined daily the number of responses collected and how many were actually valid during the data collection period;
5. Finally, 776 responses were collected between 16 June and 16 August 2019. However, 714 responses were accepted and entered into the SPSS software program. Sixty-two responses were excluded because: 28 questions relating to the “MMAS-8” were not answered, twenty-three responses did not have a chronic disease, and eleven participants did not respond to half of the survey. The missing data were excluded from the study.

(e) Data Quality Control

To increase quality control in terms of data collection and avoidance of redundancy, patients were prevented from filling out more than one questionnaire form. The first measure was verified verbally with patients by asking whether they had previously completed the same questionnaire. Secondly, as an added measure, the data collector wrote a temporary code on the survey paper with a pencil, which contained a sequential number with a dash (001- to 500-), followed by the unique patient number as per their medical records (for example, 032-patient number). These combined values were arbitrary and did not indicate the patient’s identity, which remained anonymous. This process was mainly intended to ensure that duplicates entries were disqualified even before any data entry had taken place. Once the data quality was verified, the investigator erased the patient’s number from the questionnaire form.

### 2.3. IRB Approval (Exemption/Non-Human Subject Criteria)

Secondary data analysis of the 2015 National Consumer Survey on the Medication Experience and Pharmacists’ Roles falls under Exemption #4 of Exempt of Human Subjects Research [48], which covers “research involving the study of existing data, in which information is recorded in such a way that subjects cannot be identified”. The 2015 National Consumer Survey data were anonymously collected by the University of Minnesota, College of Pharmacy. 

Likewise, the developed survey for the Oman dataset assured that data were collected anonymously. Ethical approval was obtained from the Diwan of Royal Court Medical Services’ Research Ethics Committee in Oman. A standard consent form from the Diwan of Royal Court Medical Services was used to obtain patients’ permission before filling out the survey form (Supplementary Material 3: Consent Forms).

According to the Institutional Review Board (IRB) information in the investigator’s manual on page 16 [49], a quality improvement study is not considered human subject research. As previously mentioned, data related to Oman were collected within the investigator’s workplace at the Medical Services of Diwan of the Royal Court. The data analysis and findings for the Sultanate of Oman dataset were used to improve methods for better medication adherence. Therefore, it is appropriate to consider this study as a quality improvement project. According to the Human Research Determination Form 88, Omani data collection was deemed a de-identified dataset.

### 2.4. Study Measures (Variables)

This study aimed to describe the association between medication experiences and beliefs (independent categorical variable) and self-reported medication adherence (categorical dependent variable) in patients with chronic diseases in two different societies: the USA and the Sultanate of Oman. The following variables were applied.

(a) Chronic Diseases

Ten items were used to measure the numbers of health problems and chronic diseases.

- Have you ever had any of the following chronic diseases, either now or in the past?

NOYesHeart disease?

Diabetes?

Breathing problems?

Arthritis?

Cancer?

Stroke?

Obesity?

Hypothyroidism?

High Cholesterols Level

Others?

- Please list, if any, other chronic diseases you may have and not mentioned above:

(b) Medication Adherence

The Morisky Medication Adherence Scale (MMAS-8) [44,45,46,47] was used to measure medication adherence. The MMAS-8 ranges of scoring were from 0 to 8, with a 3-level Likert scale as follows:


**Medication Adherence**

**Score**
Low adherence0 to less than 6Moderate adherence6 to less than 8High adherence8

Response categories for the MMAS-8 are yes/no for each item with dichotomous responses and a 5-point Likert response for the last item. Permission to use the MMAS-8 was accepted and the Morisky License Contract and Copyright Agreement was signed.

The Morisky Medication Adherence Questionnaire (MMAS-8)
**1.**Do you sometimes forget to take your pills?**Yes****No****2.**People sometimes miss taking their medications for reasons other than forgetting. Thinking over the past two weeks, were there any days when you did not take your medicine?

**3.**Have you ever cut back or stopped taking your medicine without telling your doctor because you felt worse when you took it?

**4.**When you travel or leave home, do you sometimes forget to bring along your medicine? 

**5.**Did you take all your medicine yesterday?

**6.**When you feel like your symptoms are under control, do you sometimes stop taking your medicine?

**7.**Taking medicine every day is a real inconvenience for some people. Do you ever feel hassled about sticking to your treatment plan?

**8.**How often do you have difficulty remembering to take all your medicine?



The use of the MMAS diagnostic adherence assessment instrument is protected by U.S. copyright and trademark laws. Permission for use is required. A license is available from Morisky Medication Adherence Research, LLC., Donald E. Morisky, ScD, ScM, MSPH, MMAR, LLC, 294 Lindura Ct., Las Vegas, NV 89138; dmorisky@gmail.com.

(c) Medication Experiences and Beliefs [50,51,52,53,54,55,56].

Three items are intended for measuring medication experiences and beliefs. These are “medicines are a life-saver”, “medicines are a burden”, and “medicines do more harm than good”, which are assessed using a 4-point Likert scale, ranging from strongly disagree to strongly agree. If patients choose agree or strongly agree, this is considered as patients having “agreed” to the statement, and if patients choose disagree or strongly disagree, this is considered as “disagree”.

(d) Demography and other factors (age, gender, ethnicity or race, and education). Each factor was analyzed separately to measure its effect on medication adherence:

Demographics: age, gender (male/female), education (less than high school/high school and higher), and race and ethnicity (white/non-white).

Race and ethnicity questions used in the Omani survey: In Oman, it is not acceptable to ask such questions. Additionally, there are different perceptions of white skin color between the two populations. A person who may be considered white in Oman may not be regarded as white in the U.S.

### 2.5. Data Analysis Plan

#### 2.5.1. Descriptive Statistics

Tables providing a summary of the study variables for the USA and the Sultanate of Oman are included. Additionally, line charts illustrating the low medication adherence of participants for the different chronic disease types and the three variables of medication experiences are presented.

#### 2.5.2. Addressing Major Research Questions

Chi-square analysis with *p*-values of less than 0.05 was conducted via SPSS software to describe the associations between the outcome of interest (low medication adherence (MMAS-8 < 6)) and medication experience variables in chronic diseases.

Thus, for each research question, the analysis was as follows:

- Do medication experiences and beliefs affect medication adherence? The three variables were used to measure medication experiences and the mean Morisky Medication Adherence Scale (MMAS-8) score was used to measured medication adherence.
  Chi-square analysis with *p*-values of less than 0.05 was also conducted to describe the relationship between the medication experiences and beliefs and the outcome of interest (low medication adherence (MMAS-8 < 6)) in chronic disease participants.
  Note: The participants’ opinions varied for each statement, between agreeing or disagreeing, whether their medication adherence was low or high. This study focused on the low medication adherence participants (MMAS-8 scores from 0 to less than 6).
- Do relationships between medication adherence and medication experiences and beliefs vary from one nation to another? A simple comparison between the percentages of the Omani and U.S. populations’ medication adherence based each country’s medication experiences and beliefs statement.

## 3. Results

### 3.1. Descriptive Findings

Descriptive results for medication experiences and beliefs, chronic diseases, medication adherence, age, gender, and educational level for U.S. and Omani participants are summarized in Table 1 below. This table shows descriptive results for patients who had at least one chronic disease.

Medication adherence differed by only 4% between the two countries, in favor of the United States. The low medication adherence rate for the Omani participants was 4% higher than for the U.S. participants, while the medium-to-high medication adherence rate for the Omani participants was 4% lower than for the U.S. participants. Gender and educational level data were not homogenous between the two populations. The number of females was higher in the U.S., while in Oman the number of females was equal to males. In Oman, nearly two-thirds of the participants were not high school graduates, while only 2% of the U.S. participants were not high school graduates.

Around 90% of the U.S. and Omani participants agreed that medication is a life-saver. Nearly 60 percent of the U.S. participants believed that medication is a burden, and 37% believed that medicine does more harm than good. The opposite was the case for Omani participants—71% believed it was not a burden and only 17% thought medication does more harm than good.

### 3.2. The Association of Medication Experiences and Beliefs with Low Medication Adherence

**Figure 3:**
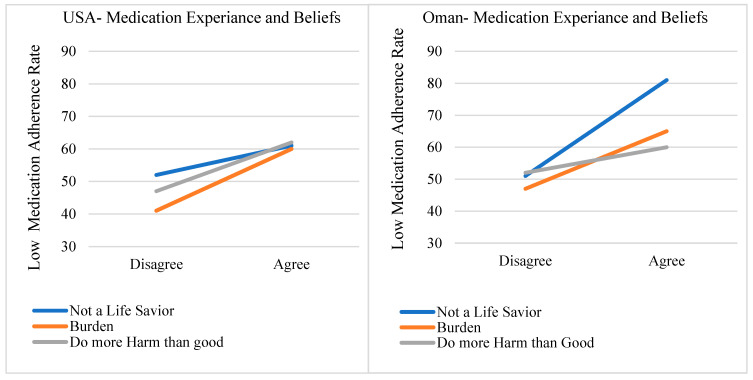
The effects of medication experiences and beliefs on the low medication adherence rates of U.S. and Omani participants.

Chi-square analysis with *p*-values of less than 0.05 was conducted to describe the relationship between the medication experience and beliefs and the outcome of interest (low medication adherence (MMAS-8 < 6)) in chronic disease participants. The findings from this analysis of the U.S. and Omani participants for patients taking at least one chronic disease medication are presented in Table 2 and illustrated with line charts in Figure 3 on the next page.

Table 2 and Figure 3 show a decrease in the low medication adherence rate in patients with positive medication experiences and beliefs (x-axis: disagree) in both countries. However, the percentage differences in the low medication adherence rates between those who “agree” and those who “disagree” differed in the two datasets.

The U.S. participants had the highest difference in low medication adherence rates, with the belief that “medication is a burden”, followed by “medication is more harmful than good”, and lastly that “medicine is life-saving”. The percentage differences in the low medication adherence rates were 19%, 15%, and 9%, respectively, in favor of positive medication experiences and beliefs.

In the Omani respondents, the highest differences in the low medication adherence rates were with the belief that “medication is a life-saver”, followed by “medication is a burden”, and lastly that “medication does more harm than good”. The percentage differences in the low medication adherence were 30%, 18%, and 8%, respectively, in favor of the positive medication experiences and beliefs.

## 4. Discussion

Medication experiences and beliefs influence people’s attitudes towards medications, and most probably influence their daily medication-taking behavior. Several studies have emphasized the importance of the necessity of taking a medication to treat a disease and to maintain or improve health, as well as the concerns about the possible harmful effects related to improving adherence [51,52,53,54,55,56,57,58,59,60,61,62,63,64]. 

Both the U.S. and Omani groups highly agreed that medication is a life-saver; this observation was consistent with other studies, which showed that stronger necessity beliefs were associated with better medication adherence [21,57,58,59,61,63,64].

Although there is a direct association between medication experiences and beliefs and medication adherence, this association has different implications for different population groups. In the Omani group, participants who believed that “medication is a life-saver” had a much lower percentage of low medication adherence, and the participants who believe that “medication does more harm than good” had a little higher percentage in the low medication adherence group. This was consistent with a previous study done in Oman on 215 hypertensive patients and their responses to the BMQ, which showed that participants with stronger beliefs about medication necessity were twice as likely to have high adherence, and participants who were concerned about their medication were about one-third as likely to have high adherence [21]. This was also in line with another study done in Italy on 427 patients with chronic diseases (depression, asthma, diabetes, and cardiac disease) and their responses to the BMQ, which showed that participants who had high necessity and low concerns, representing nearly 60% of the participants, reported the highest adherence to medication. Those who had low necessity and high concern, representing only 4% of the participants, reported the lowest adherence rates [57].

More than 90% believed that “medication is a life-saver” in the U.S. group; however, this did not decrease their low medication adherence rate as much as when they believed that “medication is not a burden”. Moreover, as they had more concerns that “medication does more harm than good”, their low medication adherence rate increased, which was also observed in other studies that showed that stronger concern beliefs were associated with decreased medication adherence in chronic disease patients [58,60,62,63]. A study in the UK of 600 out-patients with rheumatoid arthritis showed that 74% of respondents agreed or strongly agreed that their arthritis medications were necessary for their health and 47% were concerned about potential adverse consequences. This study also showed that non-adherent participants had higher concern scores than adherent participants [58]. Additionally, a cross-sectional survey conducted with 1871 participants with inflammatory bowel disease in the UK showed that 29% of the participants had low medication adherence, which was associated with doubts about personal need for taking maintenance therapies (odds ratio (OR) = 0.56; 95% confidence interval (CI): 0.48–0.64; *p* < 0.001) and concerns about potential adverse effects (OR = 1.66; 95% CI: 1.42–1.94; *p* < 0.001) [64].

This study’s findings showed that more positive medication experiences and beliefs were observed in the Omani group than the U.S. group. The finding that having the belief that “medication is a life-saver” was associated with better medication adherence in Oman than the U.S. group may be due to the strong religious beliefs that urge Muslims to seek treatment and rely on God for healing. Alternatively, it may be due to the trust of using Western medicines compared to the herbal medicines that people used to use in the past decades, and how their average life expectancy has increased accordingly. Further studies are needed to investigate these issues.

Perhaps the reason why two-thirds of the Omani groups believed medication is not a burden may firstly be because Omani citizens have free access to public healthcare and free medication, which is not true in the U.S., where financial difficulties may be one of the major burdens [14,65]. Secondly, this may be due to family solidarity and Islamic religious orders in Oman, which urge Muslims to take care of their parents, especially when they are getting older. Islam also urges kinship ties, especially between siblings and close relatives. More studies are needed to verify whether family or religious support led to this belief.

Furthermore, one of the possible justifications as to why only 17% of the Omani group believed that medication does more harm than good compared to 37% of the U.S. group could be due to educational level. Nearly two-thirds of Omani participants were not high school graduates and 23% were illiterate, compared to the United States, where only 2% were not high school graduates and no participants were illiterate. A lack of information about the side effects and adverse reactions that drugs may cause or the medicine’s effectiveness may reinforce this belief. Additionally, the high network cost and lack of network availability in some areas in Oman may be among the reasons that led to disapproval of this belief due to the individual’s inability to search for extra information about their medicines. Alternatively, the healthcare providers may not inform the patient of such information about their medication (certain side effects or adverse reactions) to prevent non-adherence. Further studies are needed to investigate these variables.

This study showed how each belief has a different effect on medication adherence and how their effects differ from one community to another. Although more than 90% believe that medication is a life-saver in the U.S. group, this did not increase their adherence as much as their belief that medication is not a burden. Meanwhile, in Oman, the belief that medication is a life-saver greatly affects medication adherence.

Thus, listening to the patients’ medication experiences and beliefs and checking their most vital concerns regarding medication will help providers to tailor their medications to increase their adherence.

### Study Limitations

The major limitation of this comparison study was using convenience sampling in the Omani dataset, which might not represent the entire population, as well as being limited by the cross-sectional nature of the U.S. dataset. However, this study is useful as a preliminary and exploratory investigation into predictors of how each belief affects adherence in different societies and provides a good starting point for longitudinal studies in this area.

Additionally, the small sample size of the Omani dataset may not have offered sufficient statistical power to determine the impacts of the investigated medication adherence variables. Another limitation is that the Omani survey was conducted in the waiting area while the patients were waiting for their appointment. This may have affected their answers due to time limits. The U.S. data may be more reliable due to the larger sample size and because it was conducted via email. Omani participants were in a stressful situation, as they were waiting for their appointment or collecting their medication.

Another limitation was that there was no reliability and validity study for the Arabic translation for the three medication experiences and belief statements.

## 5. Conclusions

Medication adherence for chronic conditions remained low. The impacts of medication experiences and beliefs affect medication adherence and differ in the two groups. The belief that “medication is a life-saver” had a significant effect on the Omani group (30%) and little effect on the U.S. group (9%) in increasing medication adherence. While nearly twice the number of U.S. participants as Omani participants believed that “medication is a burden”, both had a nearly 20% higher medication adherence in participants who believe that medication is not a burden. In contrast, only 17% of Omani believed that “medication does more harm than good”, whereas only 37% of U.S. participants believed this; medication adherence rates in the Omani group were 8% higher and in the U.S. group were 15% higher in participants who disagreed with this statement compared to participants who agreed.

Thus, in the Oman group, the statement that “medication is a life-saver” had a more significant effect on medication adherence in patients with chronic disease than “medication is a burden”. The statement that affected adherence the least was “medication does more harm than good”. In the U.S. group, the statement that “medication is a burden” has a greater effect on medication adherence than “medication does more harm than good”, while that with the lowest impact was “medication is a life-saver”. 

This study showed that a patient’s medication experiences play an essential role in medication adherence. These expereinces play a significant role in how patients perceive health education and medication counseling, affecting the extent of their acceptance of the treatment. An improved understanding of the relationships between patients’ medication experiences, disease- and treatment-related problems, and efficient provider–patient communication will lead to more effective ways to improve adherence. Therefore, proper communication between patients and healthcare providers based on the patient’s medication experiences and beliefs will substantially improve medication adherence.

### 5.1. Study Implications

This study supports the use of medication experiences and belief statements in verifying low medication adherence in chronic diseases. Patient medication experiences are focused on patient-centered care and improving the quality of pharmacy practice. This approach has important implications for healthcare providers in evaluating patients’ medication experiences and beliefs to improve medication adherence, taking into consideration their backgrounds, such as their culture and religion.

This approach can be applied as a starting point by asking three simple questions to understand which beliefs affect the patient the most, and is a good start for getting more details about the medication experiences that affect their adherence the most. This tool will allow healthcare providers to give patients the most appropriate and accurate interventions and guide them on customizing patient counseling and health education to overcome their non-adherence.

### 5.2. Future Research

This preliminary study’s findings is a good start for further research, such as measuring which beliefs have greater effects in different societies and which variables affect each belief the most (e.g., religion, culture, or society traditions). Future study is also needed to identify the associations between social support and religious beliefs and medication experiences and beliefs in different societies.

To address potential implications, a longitudinal study should be conducted for a specific chronic disease, whereby measure medication adherence is measured along with medication experiences and beliefs before and after the intervention (such as with counseling, changing dosage regimen, or overcoming certain side effects). Additionally, a study could be conducted with different medication adherence measurement methods to check whether they show the same results.

Moreover, this study will encourage more researchers to establish various methods for improving patient–provider communication skills to increase adherence by evaluating patient drug experiences and beliefs.

Future research is also needed to evaluate the validity and effectiveness of medication experiences and beliefs in actual practice, reliability and validity study for the Arabic translation of the three medication experience and belief statements.

## Figures and Tables

**Table 1:** Summary of the study variables for the USA and Oman.

Characteristics	Description	USA (*n* = 13,731)	Oman (*n* = 687)
		*n* (%)	*n* (%)
Chronic Disease Types	Heart disease	1780 (13%)	445 (65%)
	Diabetes	3051 (22%)	405 (59%)
	Arthritis	6956 (51%)	172 (25%)
	Breathing problems	6241 (46%)	87 (13%)
	Obesity	7546 (55%)	144 (21%)
	Stroke	618 (5%)	16 (2%)
	Cancer	1740 (13%)	20 (3%)
	Hypothyroidism	-------	114 (17%)
	Other chronic diseases	-------	97 (14%)
Medication Adherence	Low Adherence	6129 (52%)	351 (55%)
	Medium to High Adherence	5625 (48%)	290(45%)
Medication Experiences and beliefs			
a. Medicines are a life-saver	Disagree	1133 (8%)	75 (11%)
	Agree	(92%)	615 (89%)
b. Medicines are burden	Disagree	5570 (41%)	477 (71%)
	Agree	8161 (59%)	195 (29%)
c. Medicines do harm more than good	Disagree	8599 (63%)	552 (83%)
	Agree	5132 (37%)	116 (17%)
Age	20s	2056 (14.9%)	42 (6.5%)
	30s	2373 (17.3%)	120 (18.5%)
	40s	2293 (16.7%)	167 (25.8%)
	50s	2806 (20.4%)	176 (27.2%)
	60s	2805 (20.4%)	102 (15.8%)
	70s	1173 (8.5%)	35 (5.4%)
	80s	212 (1.5%)	5 (0.8%)
	90s	13 (0.1%)	------
Gender	Male	3839 (28%)	331 (50%)
	Female	9892 (72%)	329 (50%)
Educational Level	Less than a High School Graduate	336 (2%)	404 (61%)
	High School Graduate and Higher	(98%)	258 (39%)

**Table 2:** The effect of medication experiences and beliefs on the low medication adherence rates of U.S. and Omani participants.

Low Medication Adherence				
		USA (Total *n* = 11,754) Oman (Total *n* = 561)	USA (Total *n* = 11,754) Oman (Total *n* = 547)	USA (Total *n* = 11,754) Oman (Total *n* = 542)
		Not a life-saver *n* (%)	Burden *n* (%)	Does more harm than good *n* (%)
USA	Disagree	5655/10974 (52%)	2001/4914 (41%)	3590/7629 (47%)
	Agree	474/780 (61%)	4128/6840 (60%)	2539/4125 (62%)
Oman	Disagree	256/504 (51%)	184/388 (47%)	237/459 (52%)
	Agree	46/57 (81%)	104/159 (65%)	50/83 (60%)

## Data Availability

Not applicable.

## Supplementary Materials

The following are available online at https://www.mdpi.com/2226-4787/9/1/31/s1, Supplementary material 1: Developed Survey; Supplementary material 2: Arabic Translated Survey; Supplementary material 3: Consent forms (English and Arabic).
