# Supplementary material for: The Association between Medication Experiences and Beliefs and Low Medication Adherence in Patients with Chronic Disease from Two Different Societies: The USA and the Sultanate of Oman
Source: Pharmacy (Basel). 2021 Feb 3;9(1):31. doi: 10.3390/pharmacy9010031 (PMC7931077; doi:10.3390/pharmacy9010031)
Supplement: Supplementary file 1 [file pharmacy-09-00031-s001.pdf]

Supplementary material 1
Developed Survey

Sr No:______

Patient's Medication Compliance
Improve Patients' Compliance to Chronic Disease Medications Questionnaire Survey

The purpose of this study is to learn more about people's medication compliance, i.e., when patients take medication as prescribed by their doctors. The result of this study will help healthcare providers to deliver better care services for their patients.

You're taking the time to complete the following survey would be appreciated. It should take about 1 5minutes of your time. Be assured that all answers you provide will be kept in restricted confidentiality. All participants in the survey will remain anonymous. If you have any questions about the study, please do not hesitate to email us at ibrah122@umn.edu.

Your Eligibility to Participate

This survey tries to quantify the degree in which patients' compliance to medications and is only intended for patients with chronic diseases. If this survey is not relevant to your medical conditions, you may please disregard it and return the paper to the medical personnel.

To verify your eligibility, please answer the two following questions:

1. Do you take prescription drugs for any chronic diseases such as heart problems, diabetes, arthritis, or breathing problems …etc.?

□ No □ Yes

2. Have you filled out this questionnaire before?

□ No □ Yes

If you are taking chronic disease medications and have not completed this survey before, you may proceed to the following questions.

A. Your Thoughts About Medication

3. Please indicate to what extent you agree or disagree with each of the following statements. There is no right or wrong answer; we are interested in your personal perception regarding medication.

The statement	Strongly Disagree	Disagree	Agree	Strongly Agree
Medicines are a lifesaver.	□	□	□	□
Medicines are a burden.	□	□	□	□
Medicines do more harm than good.	□	□	□	□
Your **physician** explains to you how to take your medication in a difficult way that you cannot understand	□	□	□	□
The **pharmacist** explains to you how to take your medication in a difficult way that you cannot understand	□	□	□	□

B. Questions About Your Health

4. Do you sometimes forget to take your pills?

   □ No □ Yes

5. Thinking over the past two weeks, were there any days when you did not take your medicine?

   □ No □ Yes

6. Have you ever cut back or stopped taking your medicine without telling your doctor because you felt worse when you took it?

   □ No □ Yes

7. When you travel or leave home, do you sometimes forget to bring along your medicine?

□ No □ Yes

8. Did you take all your medicine yesterday?

□ No □ Yes

9. When you feel like your symptoms are under control, do you sometimes stop taking your medicine?

□ No □ Yes

10. Do you feel hassled about sticking to your daily medicine schedule?

□ No □ Yes

11. How often, if at all, do you have difficulty remembering to take all your medicine?

12. Have you ever had any of the following chronic diseases, either now or in the past?

	No	Yes
Heart disease	◻	◻
Diabetes	◻	◻
Breathing problems (*Such as asthma, COPD…etc)*	◻	◻
Arthritis	◻	◻
Cancer	◻	◻
Stroke	◻	◻
Obesity	◻	◻
Hypothyroidism	◻	◻
High Cholesterols Level	◻	◻
Others	◻	◻

13. Please list, if any, other chronic diseases you have not mentioned above:

-- -----
----

14. How many health problems, if any, do you **currently** have? (choose only 1 answer)

◻ One ◻ Two ◻ Three ◻ Four ◻ Five ◻ Six ◻ Seven ◻ More than seven

15. How do you rate your overall health?

◻ Excellent ◻ Good ◻ Fair ◻ Terrible

16. When have you been hospitalized?

□ This year □ Last year □ Both years □ Neither

17. How many of the following medications, if any, you are currently taking every day?

_____ prescription drugs

_____ over-the-counter drugs

_____ herbal supplements

18. How many times per day do you take all your current chronic disease medication? (choose only 1 answer)

□ One dose daily

□ Two doses daily

□ Three doses daily

□ Four doses daily

□ Five doses daily

□ More than five doses daily

C. General Questions About You

20 What is your gender?

□ Male □ Female

21 What is your current age in years? ____________

22 Which of the Diwan of Royal Court Medical Centers do you usually take your chronic diseases medication supply ?

□ Muscat

□ Salalah

23 Where do you currently live? (choose only one answer)

- □ Muscat Governorate
- □ Dhofar Governorate
- □ Ad Dakhiliyah Governorate
- □ Ad dhahirah Governorate
- □ Al Batinah Governorate
- □ Al Buraimi Governorate
- □ Al Wusta Governorate
- □ Ash Sharqiyah Governorate
- □ Musandam Governorate

24 What is your highest level of education? (choose only one answer)

- □ Illiterate
- □ Ability to read and write only
- □ Less than High School Graduate
- □ High School Graduate
- □ Some College, no degree /diploma
- □ Bachelor's Degree
- □ Master's Degree
- □ Professional Degree
- □ Doctoral Degree

End of the Survey

Supplementary material 2
Arabic Translated Survey

إستبيان لتحسين التزام المرضى بتناول أدوية الأمراض المُزمنة

الغرض من هذه الدراسة هو معرفه المزيد عن مدى التزام المرضى بتناول الأدوية ، اي عندما يتناول المرضي الأدوية علي النحو الموصوف من قبل أطبائهم . ستساعد نتيجة هذه الدراسة مقدمي الرعاية الصحية علي تقديم خدمات رعاية أفضل للمرضي .

مشاركتك في الإجابة على أسئلة هذا الاستبيان والتي قد تأخذ من وقتك حوالي 15 دقيقة سيكون موضع تقدير منا . ونؤكد لك ان جميع الإجابات التي ستُقدمها سيتم التعامل معها بمنتهى السرية ، كما أن جميع المشاركين في هذه الدراسة ستبقى هوياتهم مجهولة. إذا كان لديك اية اسئلة حول الدراسة ، فمن فضلك لا تتردد في مراسلتنا علي ibrah122@umn.edu .

مدى أهليتك للمشاركة في هذا الإستبيان

المرض المُزمن هو مرض دائم يأتي مع الوقت ويتقدم بشكل بطيئ ويستمر لفترة طويلة غالباً لمدة ثلاثة أشهر أو أكثر.

للتحقق من أهليتك للمشاركة في هذا الاستبيان، يُرجى الاجابة علي السؤالين التاليين:

1. هل تاخذ وصفات طبية لأي من الأمراض المزمنة كالضغط ، السكري ، التهاب المفاصل، أو مشاكل في التنفس ... الخ؟

□ لا　　□ نعم

2. هل قمت بتعبئة هذا الاستبيان من قبل؟

□ لا　　□ نعم

إن كنت تتناول أدوية الأمراض المزمنة ولم يسبق لك تعبئة هذا الاستبيان ،

فيمكنك الانتقال للإجابة على الاسئلة التالية .

أولاً : آراؤك حول الدواء والاستشارات الدوائية

3. يُرجى الإشـــارة إلى أي مدى أنت توافق أو لا توافق على كل عبارة من العبارات التالية . ليس هناك جواب صائب أو خاطئ ، فنحن هنا مهتمون إلى معرفة وجهة نظرك الشخصية حول الأدوية .

العبارة	أُعارض بشدة	أُعارض	أوافق	أوافق بشدة
الأدويـة مُنقذة للحيــاة	◻	◻	◻	◻
الأدويـة هي عِبْء	◻	◻	◻	◻
الأدوية تضُر أكثر مما تنفع	◻	◻	◻	◻
يشرح لك **الطبيب** كيفية تناول الدواء بطريقة صعبة لا يمكنك فهمها	◻	◻	◻	◻
يشرح لك **الصيدلي** كيفية تناول الدواء بطريقة صعبة لا يمكنك فهمها	◻	◻	◻	◻

ثانياً : أسئلة تتعلق بصحتك

السؤال من 4 الى 11(اسئلة مقياس مروسكي لالتزام بالأدوية (MMAS-8)):

الترجمة العربية لـ MMA_8 محمية بموجب حقوق النشر. للمزيد من المعلومات أرجو الأتصال:

The use of the MMAS diagnostic adherence assessment instrument is protected by U.S.
copyrighted and trademarked laws. Permission for use is required. A license is available from -
MORISKY MEDICATION ADHERENCE RESEARCH, LLC., Donald E. Morisky, ScD, ScM, MSPH, MMAR, LLC, 294 Lindura Ct., Las Vegas, NV 89138; dmorisky@gmail.com

12. هل تعاني حالياً أو سبق أن عانيت من الأمراض المزمنة التالية ؟

	لا	نعم
أمراض القلب (مثال: الضغط)	◻	◻
السكري	◻	◻
مشاكل التنفس (مثال : الربو ، الإنسداد الرئوي المزمن .. الخ)	◻	◻
إلتهاب المفاصل	◻	◻
السرطان	◻	◻
السكتة الدماغية	◻	◻
السمنة	◻	◻

نقص هرمون الغدة الدرقية	□	□
إرتفاع مستوى الكولسترول	□	□
أخرى	□	□

13. يُرجى ذكر الأمراض المزمنة الأخرى ، إن وُجدت ؟

----

14. كم عدد الأمراض المزمنة التي لديك حالياً ، إن وجدت؟ (إختر إجابة واحدة فقط)

□ واحدة □ إثنتين □ ثلاثة □ أربعة □ خمسة □ ستة □ سبعة □ أكثر من سبعة

15. كيف تقيم صحتك بصفة عامة ؟

□ ممتازة □ جيدة □ مقبولة □ سيئة جداً

16. بالنظر إلى السنتين الماضيتين ، هل تم تنويمك في المستشفى ؟ (إختر إجابة واحدة فقط)

□ هذه السنة فقط □ السنة الماضية فقط □ في كلتا السنتين □ لم يتم تنويمك

17. كم **حبة** تتناول حالياً في **اليوم الواحد** من الأدوية التالية ، إن وجدت ؟

______ أدوية وصفات طبية

______ أدوية بدون وصفات طبية

______ مكملات عشبية

18. كم **مرة** في اليوم تتناول أدويتك الخاصة بالامراض المزمنة التي تعاني منها حالياً؟ (إختر إجابة واحدة فقط)

□ مرة واحدة في اليوم

□ مرتين في اليوم

□ ثلاث مرات في اليوم

□ أربع مرات في اليوم

□ خمس مرات في اليوم

□ أكثر من خمس مرات في اليوم

ثالثا: أسئلة عامة عنك

19. ما هو نوع جنسك ؟

□ ذكر □ أنثى

20. كم عمرك بالسنوات ؟ ___________

21. ما هو المركز الصحي التابع لديوان البلاط السلطاني الذي عادة ما تستلم منه أدويتك الخاصة بالامراض المزمنة؟ (حدد إجابة واحدة فقط)

□ مسقط

□ صلالة

22. أين تسكن حالياً ؟ (إختر إجابة واحدة فقط)

□ محافظة مسقط
□ محافظة ظفار
□ محافظة الداخلية
□ محافظة شمال الباطنة
□ محافظة جنوب الباطنة
□ محافظة شمال الشرقية
□ محافظة جنوب الشرقية
□ محافظة مسندم
□ محافظة البريمي
□ محافظة الظاهرة
□ محافظة الوسطى

23. ما هو مستواك التعليمي ؟ (إختر إجابة واحدة فقط)

□ لا تقرأ ولا تكتب
□ تقرأ وتكتب فقط
□ أقل من شهادة دبلوم التعليم العام
□ شهادة دبلوم التعليم العام
□ بعض التعليم الجامعي ، بدون شهادة جامعية
□ درجة البكالوريوس/شهادة جامعية
□ درجة الماجستير
□ درجة مهنية
□ درجة الدكتوراه

~نهاية الاستبيان ، شاكرين لك تعاونك معنا~

Supplementary material 3
Consent forms (English and Arabic)

Sultanate Oman
Diwan of Royal Court
Directorate of Medical Services
DG Office (Research)

Consent

Dear participant,

We are conducting a study about:

Improve Patients' Adherence to Chronic Disease Medications.

This study will add to the body of knowledge.

You are kindly invited to participate in this study because you meet the study criteria. If you agree to participate in this study, you will be asked ***to fill a Questionnaire Survey.***

Any information that is obtained in connection with this study will remain confidential and will not be a part of a record that can be linked to you.

Your participation in this study is voluntary. Your decision whether or not to participate will not affect your treatment at the Diwan of Royal Court. When you decide to participate, you are free to withdraw your consent and to discontinue participation at any time without affecting current or future care. The researchers do not anticipate any physical or emotional harm to you from this study. *(if harms are anticipated, please describe them clearly).*

The Institutional Review Board of the Diwan of Royal Court Medical Services has reviewed and approved this research.

We are grateful for your participation in this study.

If you have any question related to this study, we can be reached at

Email: hhmrahbi@diwan.gov.om or *Ibrah122@umn.edu*.

If you agree to participate in this study, please sign/indicate below

Written consent		Verbal consent	
Date:		Date:	
Name of participant:		Name of person obtaining consent:	
Signature:		Signature:	

سلطنة عمان
ديوان البلاط السلطاني
المديرية العامة للخدمات الطبية
مكتب المدير العام (البحث الطبي)

موافقة

نحن نقوم ببحث يختص بدراسة: **تحسين التزام المرضى بأدوية الامراض المزمنه**
وبسبب إستيفائك لكافة معايير هذه الدراسة ، يرجى منك تكرما أن تشارك بها. وفي حال قبولك المشاركة ، سوف يُطلب منك **تعبئة استبيان استقصائي** .
مشاركتك في هذه الدراسة هي اختيارية. كما أن قرارك بالمشاركة أو عدمها لن يؤثر على علاجك الذي تتلقاه من ديوان البلاط السلطاني. في حال قررت المشاركة ثم تراجعت عن ذلك فإنه يمكنك أن تسحب/ تسحبي قرارك وتوقف/ توقفي مشاركتك في أي وقت بدون أن يؤثر ذلك على الرعاية التي تتلقاها/ تتلقينها حاليا أو في المستقبل. إن الباحث لا يتوقع أن يحدث لك أي ضرر مادي أو معنوي من جراء مشاركتك.
إن هذه الدراسة قد تم مراجعتها والموافقة عليها من جانب قسم المراجعة ولجنة الأخلاق المهنية بالمديرية العامة للخدمات الطبية/ ديوان البلاط السلطاني. و نحن ممتنين لمشاركتك في هذه الدراسة.
إذا كان لديك أي سؤال يتعلق بهذه الدراسة ، فإنه يمكنك التواصل على الرقم : 24213265
الإيميل: hhmrahbi@diwan.gov.om او Ibrah122@umn.edu

في حال موافقتك على المشاركة في هذه الدراسة ، الرجاء التوقيع/الاشارة أدناه:

**موافقة خطية**	**موافقة شفهية**
**التاريخ:**	**التاريخ:**
**الاسم:**	**اسم الشخص الحاصل على الموافقة:**
**التوقيع:**	**التوقيع:**

اسم الباحثة
صيدلانيه متخصصة اولى/ كاملة بنت ميلاد الغسانية
